# Integrating plot-based methods for monitoring biodiversity in island habitats under the scope of BIODIVERSA + project BioMonI: Tree monitoring in Terceira, Tenerife and Réunion Islands

**DOI:** 10.3897/BDJ.13.e158423

**Published:** 2025-06-23

**Authors:** Paulo A. V. Borges, Leila N. Morgado, Rosalina Gabriel, Rui B. Elias, Mirana Gauche, Claudine Ah-Peng, Rüdiger Otto, Lea de Nascimento, Dominique Strasberg, Nathaly Guerrero-Ramírez, Holger Kreft, José María Fernández-Palacios

**Affiliations:** 1 University of Azores, CE3C—Centre for Ecology, Evolution and Environmental Changes, Azorean Biodiversity Group, CHANGE —Global Change and Sustainability Institute, School of Agricultural and Environmental Sciences, Rua Capitão João d’Ávila, Pico da Urze, 9700-042, Angra do Heroísmo, Azores, Portugal University of Azores, CE3C—Centre for Ecology, Evolution and Environmental Changes, Azorean Biodiversity Group, CHANGE —Global Change and Sustainability Institute, School of Agricultural and Environmental Sciences, Rua Capitão João d’Ávila, Pico da Urze, 9700-042 Angra do Heroísmo, Azores Portugal; 2 IUCN SSC Atlantic Islands Invertebratte Specialist Group, Angra do Heroísmo, Azores, Portugal IUCN SSC Atlantic Islands Invertebratte Specialist Group Angra do Heroísmo, Azores Portugal; 3 IUCN SSC Monitoring Specialist Group, Angra do Heroísmo, Azores, Portugal IUCN SSC Monitoring Specialist Group Angra do Heroísmo, Azores Portugal; 4 Miharisoa Gauche, UMR PVBMT, Université de la Réunion, 7 chemin de l’IRAT, 97410, Saint-Pierre, La Réunion, France Miharisoa Gauche, UMR PVBMT, Université de la Réunion, 7 chemin de l’IRAT, 97410 Saint-Pierre, La Réunion France; 5 UMR PVBMT, Université de la Réunion, 7 chemin de l’IRAT, 97410, Saint-Pierre, La Réunion, France UMR PVBMT, Université de la Réunion, 7 chemin de l’IRAT, 97410 Saint-Pierre, La Réunion France; 6 OSU-R, Université de la Réunion, CNRS, IRD, Météo France, 15 Avenue René Cassin, CS92003, 97744, Saint-Denis, La Réunion, France OSU-R, Université de la Réunion, CNRS, IRD, Météo France, 15 Avenue René Cassin, CS92003, 97744 Saint-Denis, La Réunion France; 7 Island Ecology and Biogeography Group, Instituto Universitario de Enfermedades Tropicales y Salud Pública, Universidad de La Laguna, La Laguna, 38200, Canary Islands, Spain Island Ecology and Biogeography Group, Instituto Universitario de Enfermedades Tropicales y Salud Pública, Universidad de La Laguna La Laguna, 38200, Canary Islands Spain; 8 UMR PVBMT, Université de la Réunion, 15 Avenue René Cassin, CS92003, 97744, Saint-Denis Cedex 9, La Réunion, France UMR PVBMT, Université de la Réunion, 15 Avenue René Cassin, CS92003, 97744 Saint-Denis Cedex 9, La Réunion France; 9 Biodiversity, Macroecology and Biogeography, Faculty of Forest Sciences and Forest Ecology, University of Göttingen, Göttingen, Germany Biodiversity, Macroecology and Biogeography, Faculty of Forest Sciences and Forest Ecology, University of Göttingen Göttingen Germany; 10 Centre of Biodiversity and Sustainable Land Use, University of Göttingen, Göttingen 37077, 14, Germany Centre of Biodiversity and Sustainable Land Use, University of Göttingen Göttingen 37077, 14 Germany; 11 Ecology Department, Biology Faculty La Laguna University, 38206, La Laguna, Tenerife, Canary Islands, Spain Ecology Department, Biology Faculty La Laguna University, 38206 La Laguna, Tenerife, Canary Islands Spain

**Keywords:** local plot-based monitoring, native forest, occurrence, Réunion, Tenerife, Terceira, woody plants

## Abstract

**Background:**

Oceanic islands are globally recognised for their exceptional levels of biodiversity and endemism, often resulting from unique evolutionary processes in isolated environments. However, this biodiversity is also disproportionately threatened by anthropogenic pressures including habitat loss, invasive species and climate change. Targeted, long-term biodiversity monitoring is essential for detecting changes in these vulnerable ecosystems and providing information for conservation strategies.

The EU BIODIVERSA + project BioMonI aims at building a global long-term monitoring network specifically tailored to the pressing needs of biodiversity conservation and monitoring on islands. In BioMonI, we use a novel approach that considers mapping previous and current monitoring schemes on islands, developing a harmonised monitoring scheme for island biodiversity and mobilising existing monitoring data. We are assembling data from BioMonI-Plot, a long-term vegetation plot network to understand biodiversity and ecosystem change. It will use baseline data from three focal archipelagos (Azores, Canary Islands and Mascarenes), but we aim to mobilise data from archipelagos worldwide.

Plot-based data are a cornerstone of effective biodiversity monitoring on islands. These standardised data collections within permanent plots allow for consistent, replicable observations across temporal and spatial scales. Initiatives like the Global Island Monitoring Scheme (GIMS) highlight the value of permanent plots in capturing ecological gradients and anthropogenic disturbance patterns. Such data underpin the detection of subtle shifts in community composition, functional diversity and species distributions, which are critical for assessing the effectiveness of conservation actions and predicting future ecological scenarios.

In summary, plot-based data are indispensable for targeted and effective biodiversity monitoring on islands. They provide the empirical backbone necessary to provide information for adaptive management strategies and contribute to global biodiversity targets.

**New information:**

The BioMonI-Plot baseline data consist of 10 plots in each of the following islands: Terceira (Azores), Tenerife (Canaries) and Réunion Island (Mascarenes). As a first step, we describe the diversity and abundance of all woody species shoots with a diameter at breast height (DBH) ≥ 1 cm in each of the 10 plots of each Island. The majority of taxa belonged to the phylum Magnoliophyta, which accounted for 96.66% of the total species and subspecies, followed by Pteridophyta (2.22%) and Pinophyta (1.11%). Réunion Island exhibited the highest species richness, with 66 identified taxa, followed by Tenerife (16 taxa) and Terceira (11 taxa). Only one species, *Morellafaya*, was shared between the islands, occurring in both Terceira and Tenerife. Most of the recorded species were classified as endemic according to their colonisation status. Specifically, 32 species were endemic to the Mascarene Islands, 22 to Réunion, nine to the Azores, eleven to Macaronesia and four to the Canary Islands.

The data presented in this Data Paper provide a valuable proxy for evaluating the ecological integrity and overall habitat quality of native montane forests across three oceanic archipelagos: the Azores, Canary Islands and Mascarene Islands. By focusing on tree species as primary ecological indicators, the dataset offers insights into essential structural and compositional attributes of these ecosystems, including species richness, relative abundance and patterns of dominance.

The comprehensive species-level information contained in this dataset allows for comparisons of forest composition across islands and biogeographic regions, contributing to our understanding of insular forest dynamics, endemism patterns and conservation priorities in tropical and subtropical montane environments.

## Introduction

Oceanic islands are formed through submarine volcanic activity, primarily composed of basaltic substrates and have remained geologically isolated from continental landmasses (Whittaker et al. 2023). These islands vary in age, geographic location and degree of isolation, resulting in unique ecological characteristics and distinct biotas that are not found elsewhere (Whittaker et al. 2008, Borges et al. 2009, Whittaker et al. 2023)

Oceanic islands, despite representing a small fraction of the Earth's land surface (around 5%), contribute disproportionately to global biodiversity (Fonseca et al. 2006). They are renowned for their high levels of endemism and host a multitude of species that exhibit peculiar evolutionary trajectories and functional traits, shaped by long-term geographic isolation (Borges et al. 2009, Borregaard et al. 2016, Whittaker et al. 2023). These distinct biological assemblages make islands invaluable natural laboratories for studying ecological and evolutionary processes (Whittaker et al. 2008, Borregaard et al. 2016).

The ecological processes of immigration and extinction are fundamental to understanding species diversity on islands, with two key variables associated with these processes: island size and degree of isolation (MacArthur and Wilson 1967). In general, oceanic islands exhibit lower species richness per unit area compared to equivalent continental regions (Whittaker et al. 2023). However, the levels of endemism are higher (Kier et al. 2009) and the vulnerability of these species is greater compared to those on continents, due to the limited and unique geographical space, the specificity of their interactions with the local biotic and abiotic environment (Walter 2004, Fonseca et al. 2006) and the lower genetic variability that often characterises island populations (Frankham 1997). The process of endemism in island biotas is exclusively a result of oceanic dispersal, as these islands have never been physically connected to continental landmasses (Cowie and Holland 2006).

However, this richness comes with profound vulnerability. Islands are increasingly recognised as epicentres of biodiversity change, largely due to their sensitivity to anthropogenic pressures such as habitat destruction, invasive species and the accelerating impacts of climate change (Borges et al. 2009, Caujapé-Castells et al. 2010). Globally, 31% of priority areas for expanding protected areas are located on islands (Rodrigues et al. 2004). Amongst the biodiversity hotspots currently identified by Conservation International, nine are entirely composed of islands, including Madagascar and its surrounding islands (the Comoros, the Mascarenes and Seychelles) in the African Region. Additionally, three other hotspots contain significant portions of their biodiversity within island ecosystems: Mediterranean Basin (including the Atlantic islands of Macaronesia); as well as the Western Ghats and Sri Lanka and Sundaland in the Asia - Pacific Region (Whittaker and Fernandez-Palácios 2007, Orueta and Gena 2009).

To design networks of protected areas, accurately assess species losses or understand the processes that sustain species diversity, conservation science must take into account the spatial organisation of biodiversity (Socolar et al. 2016, Borges et al. 2018b). Understanding the relationship between locally collected monitoring data and the dynamics of regional diversity is crucial, as is understanding how the mechanisms that maintain diversity vary at local and regional spatial scales (Anderson 2018). This knowledge is essential for determining the most effective biodiversity conservation strategies at different spatial scales (McClain et al. 2011). For example, by examining changes in beta diversity, it is possible to derive a scaling factor that makes it possible to predict changes in gamma diversity, based on changes in alpha diversity (Barton et al. 2013). Furthermore, deriving beta diversity from alpha-scale survey data reveals not only the spatial organisation of biodiversity (Buckley and Jetz 2008), but also the processes that determine these patterns (Kraft et al. 2011).

Research on the diversification of communities in island biota is crucial for advancing understanding of biogeography, evolutionary biology and conservation biology (Cowie and Holland 2006, Whittaker and Fernandez-Palácios 2007, Borregaard et al. 2016). Therefore, it is essential to characterise and monitor the dynamics and responses of different taxonomic groups in order to provide information and refine conservation strategies for island biodiversity, both in the present and in the face of future environmental changes (Borges et al. 2018b, Borges 2025).

The EU Net-Biome projects ISLAND-BIODIV (2012-2015) (Borges et al. 2018a, Emerson et al. 2022) and MOVECLIM (2012-2015) (Gabriel et al. 2024) have been two landmark initiatives in addressing urgent conservation challenges faced by island ecosystems. These projects laid the groundwork for long-term biodiversity monitoring by establishing permanently georeferenced, site-based monitoring plots across various elevational and habitat gradients. These local-scale plots were designed to systematically capture spatial and temporal changes in species composition, community structure and ecosystem processes, thereby providing critical baseline data to support adaptive management and conservation planning (see also Borges et al. (2018b)). Since their inception, they have supported collaborative projects aimed at integrating knowledge on island biodiversity patterns, sustainable resource use and policy development. Building upon this infrastructure, a new long-term research initiative — SLAM (Long-Term Ecological Study of the Impacts of Climate Change in the Natural Forests of the Azores) — was launched in 2012 in the ten Terceira Island ISLAND-BIODIV plots (Borges et al. 2020, Borges 2025). This project aims to assess the impacts of key drivers of biodiversity erosion, such as climate change, invasive species and land-use transformation, on the arthropod communities inhabiting the native Azorean forests. SLAM represents one of the most consistent long-term ecological monitoring efforts in the Macaronesian Region, providing invaluable data for understanding temporal trends in species diversity and providing information for conservation strategies (Borges 2025). Using now the 30 ISLAND-BIODIV plots (10 in Terceira, 10 in Tenerife, 10 in Réunion), a new project was recently launched, the EU BIODIVERSA + project BioMonI.

The current study is the first Data Paper produced under the scope of EU BIODIVERSA + project BioMonI. The BioMonI project aims at building a global long-term monitoring network specifically tailored to the pressing needs of biodiversity conservation and monitoring on islands. Additional aims of this project are:

1) leveraging historical archives on Essential Biodiversity Variables (EBV) and Essential Ecosystem Service Variables (EESV), while developing robust biodiversity informatics tools and interoperable IT infrastructure to support data standardisation, analysis, visualisation and the effective valuation of biodiversity and ecosystem services;

2) providing optimised and standardised field sampling protocols and tested methods that combine long-term monitoring with emerging technology such as environmental DNA and remote sensing;

3) conducting targeted resurveys and establishing a network of new long-term monitoring plots;

4) scaling up the monitoring of biodiversity and ecosystem structure, functioning and services using remote sensing, macroecological modelling and future scenarios.

## General description

### Purpose

This study investigates the patterns of taxonomic composition of woody plant species in the native forests of three geographically distinct oceanic islands, each with contrasting climates: temperate (Terceira Island), subtropical (Tenerife Island) and tropical (Réunion Island). The comparison was conducted at a local scale using standardised sampling techniques (Borges et al. 2018b). By meeting these objectives, the study aims to contribute meaningfully to biodiversity research and the sustainable management of these unique forest habitats.

In a previous study, we investigated variations in species rarity, alpha, beta and gamma diversity within and between three islands (Borges et al. 2018a). In the current paper, we: i) describe the core 30 plots (10 in each island); ii) revise the taxonomic nomenclature of sampled tree species; iii) provide the distribution and abundance of all woody species shoots with a diameter at breast height (DBH) ≥ 1 cm using the best practice Darwin Core format (see GBIF dataset in Morgado et al. (2025)); iv) describe the metadata in the main text and, in the supplementary material, we provide the mean diameter at breast height (DBH > 10 cm) for the plant community in each of the 10 monitoring plots established on each island.

In doing so, we address the critical need to make biodiversity data publicly available to support long-term ecological studies and conservation planning. Specifically, our efforts contribute to overcoming two major biodiversity knowledge shortfalls: the Wallacean shortfall, by providing high-resolution species distribution data and the Prestonian shortfall, by generating standardised abundance data across spatial and temporal scales. These contributions are essential for improving macroecological inference, providing information for species conservation status assessments and guiding evidence-based policy and management decisions (Cardoso et al. 2011).

Moreover, the data here provided are to be considered the baseline data for the implementation of long-term monitoring of 30 plots (10 in each Island).

### Additional information

The islands of Terceira (Azores), Tenerife (Canary Islands) and Réunion (Mascarene Islands) host distinct plant communities, influenced by factors such as climate, topography, biogeographic history and anthropogenic activities. In fact, preserving these communities necessitates a comprehensive understanding of their unique characteristics and the challenges they face from climate change and local anthropogenic pressures.

## Project description

### Title

BIOMONI_ISLAND-BIODIV project: Biodiversity monitoring of Trees on Island ecosystems

### Personnel

Fieldwork (site selection and experimental setting): Rui B. Elias, José Maria Fernández-Palacios and Dominique Strasberg.

Fieldwork (authorisation): Azorean and Réunion Ministers of Environment. For Canary Islands, the authorisation came from the Island Council (Cabildo Insular de Tenerife).

Tree species sampling and identification: Antonio J. Pérez Delgado, Dominique Strasberg, Fernando Pereira, Jacques Fournel, José María Fernández-Palacios, Juli Caujapé‑Castells, Lea de Nascimento, Loic Cecilio, Rui B. Elias, Rüdiger Otto, Silvia Fernández Lugo.

Fieldwork assistance: Rienk Apperloo, Manuel Arechavaleta, Salvador de La Cruz, Carla Díaz, Sara Ravagni, Benito Vispo, Guillermo Sánchez, Isabel Sancibrián, Nuria Macías, Nieves Zurita (Tenerife); Anne-Marie Sadeyen, Loïc Cecilio, Noémie Mollaret, Fanny Veinante, Laura Doutre, Dominique Hoareau, Grégoire Cortial (Réunion).

Darwin Core Databases: Leila Morgado, Rosalina Gabriel and Paulo A. V. Borges.

### Study area description

Terceira Island, about 3.52 million years old, is located in the Atlantic Ocean, in the Central Group of the Azores Archipelago, at 38°43′ N and 27°12′ W, with a total area of 402.2 km². Its highest point, located in the Santa Bárbara Mountain range on the western side, reaches an elevation of 1021 m a.s.l. (above sea level) (Forjaz 2004). The Island is characterised by high relative humidity and mild temperatures with minimal seasonal fluctuations (Azevedo 2001). In the Santa Bárbara Mountain, the average annual rainfall exceeds 3400 mm and the mean temperature is 9°C (Azevedo et al. 2004).

Tenerife Island, approximately 11 million years old, is located in the Atlantic Ocean off the northwest coast of Africa, at 28.28° N, 16.15° W. It is the largest of the Canary Islands, with a total area of 2034 km². The Island's climate is influenced by the northeast trade winds, which generate a cloud layer over the northern part of the Island. This results in a climatic contrast, with the northern areas being more humid and temperate, while the southern regions are characterised by hotter and more arid conditions. At the Island's centre lies the plateau of the Las Cañadas volcanic caldera, situated at an altitude of approximately 2000 m a.s.l., with Mount Teide summit reaching 3718 m a.s.l. (Fernández‐Palacios 1992).

Réunion Island, part of the Mascarene Archipelago, is approximately 2.1 million years old. It is located in the Indian Ocean at coordinates 21°06' S, 55°31' E, covering a total area of 2512 km². The Island lies about 800 km east of Madagascar. It experiences a humid tropical climate characterised by two relatively distinct seasons (Réchou et al. 2019). The region experiences a hot and rainy season (summer), influenced by cyclonic activity that brings intense rainfall, followed by a cooler and relatively drier season (winter). Temperatures are moderate, with the average maximum temperature along the coast reaching 32.4°C during summer and the minimum in winter around 16°C. Temperature decreases with altitude and the higher regions of the Island (above 1800–2000 m a.s.l.) are prone to frequent night frosts (Badré and Cadet 1978, Réchou et al. 2019). The average annual rainfall shows a significant dissymmetry between eastern and western sides of the island due to the topography and high elevation (Réchou et al. 2019), reaching 3070 m a.s.l. (Thébaud et al. 2009).

The zones on the three Islands were selected, based on the distribution of their vegetation belts, which was predominantly comprised of endemic and native species from humid and relatively undisturbed forests. The study sites were essentially pristine on two of the Islands (Terceira and Réunion), while on Tenerife, the sites consisted of a mix of pristine and historically anthropogenically altered forests. The dominant species in Terceira included Juniperusbrevifolia(Hochst. ex Seub.)Antoinesubsp.brevifolia, *Laurusazorica* (Seub.) Franco, *Myrsineretusa* Aiton and *Vacciniumcylindraceum* Sm.; on Tenerife, they were *Ericacanariensis* Rivas-Mart., M. Osorio & Wildpret, *Ericaplatycodon* (Webb & Berthel.) Rivas-Mart. & al., *Laurusnovocanariensis* Rivas-Mart., Lousa, Fern. Prieto, E. Días, J.C. Costa & C. Aguiar, *Morellafaya* (Aiton) Wilbur, *Prunuslusitanica* L.; and on Réunion, they were *Danaisfragrans* (Lam.) Pers., *Gaertneravaginata* Poir., *Molinaeaalternifolia* Willd. and *Phyllanthusphillyreifolius* Poir. (Borges et al. 2018b).

### Design description

**Figure 1.**
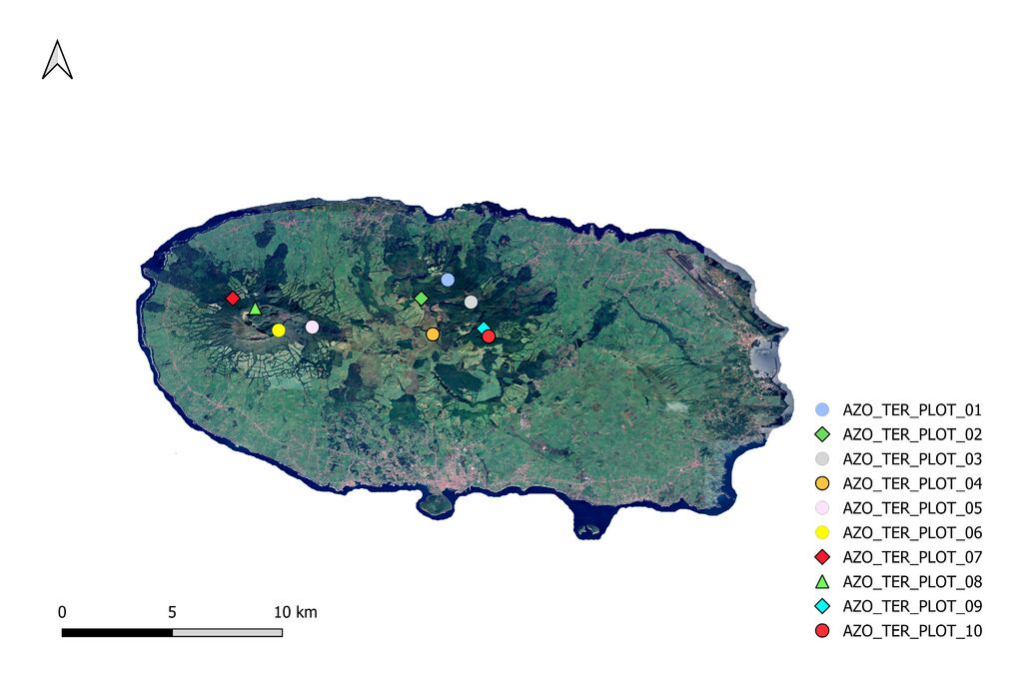
Map of Terceira Island with the sampling points (eventID). Source: HCMGIS Plugins, with modifications.

**Figure 2.**
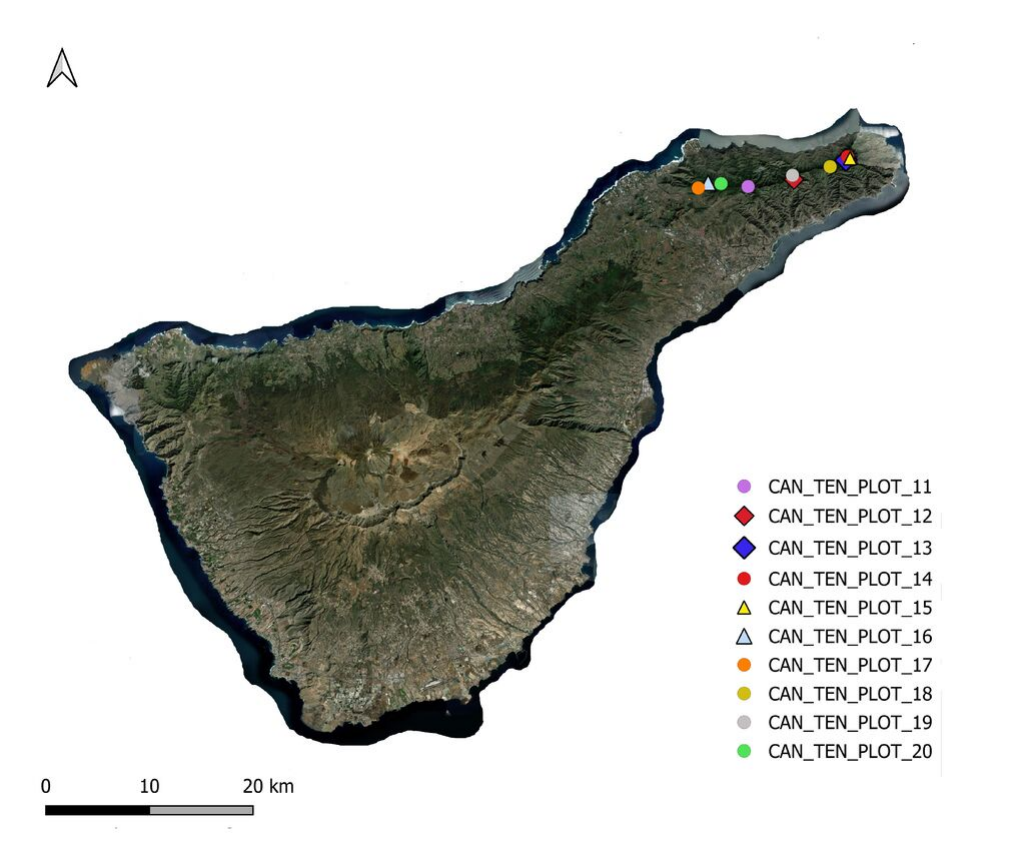
Map of Tenerife Island with the sampling points (eventID). Source: HCMGIS Plugins, with modifications.

**Figure 3.**
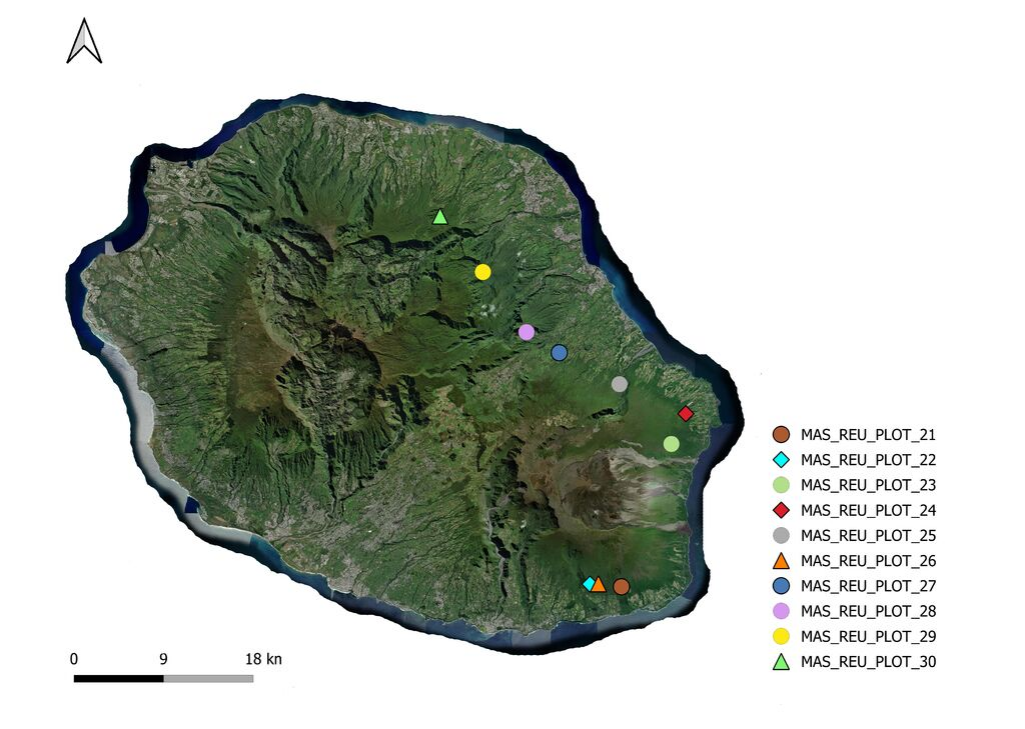
Map of Réunion Island with the sampling points (eventID). Source: HCMGIS Plugins, with modifications.

This study constitutes an inventory of woody plant species in native humid forests across three geographically distinct oceanic islands: Terceira Island (Atlantic Ocean), Tenerife Island (Atlantic Ocean) and Réunion Island (Indian Ocean). The research was conducted in 2012 and 2013 with the aim of investigating patterns in the taxonomic diversity of woody plants on these Islands. A standardised protocol was employed to identify the tree species present in ten 50 m × 50 m plots (Table 1) (Figs 1, 2, 3) within native humid forests on each Island (Borges et al. 2018a, Borges et al. 2018b). This protocol named "Whole plot plant species survey" by Borges et al. (2018b), consists of performing the following: i) complete vascular plant species survey; ii) count all woody species shoots with a diameter at breast height (DBH) ≥ 1 cm in four 5 m x 5 m square subplots located in the four corners of the main plots and iii) the calculaton of tree basal area for trees with DBH > 10 cm (Basal area [m^2^ wood at breast height (approx. 1.30 m)]) and canopy height.

In addition, tree species density was also calculated, based on the following: On each corner and in the centre, of the main (2,500 m^2^) plot, a 5 m × 5 m subplot is delimited. Every shoot with a DBH > 1 cm is counted in order to determine the density of shoots per species (expressed as the mean number of shoots per square metre and per hectare).

In a previous study, we investigated variations in species rarity, alpha, beta and gamma diversity within and between three islands (Borges et al. 2018a). In the current paper, we provide the density of trees, based in this protocol using the best practice Darwin Core format (see Morgado et al. (2025)) and, in Supplementary material, we provide the mean DBH > 10 cm for the plants in each of the 10 plots of Terceira Island (Suppl. material 1), Tenerife Island (Suppl. material 2) and Réunion Island (Suppl. material 3).

Taxonomic nomenclature was revised and updated from the initial list of species in Borges et al. (2018a) and follows the AZORES BIOPORTAL for Terceira Island, the BIOTA CANARIES for Tenerife Island and TAXREF (2025) for Réunion Island.

### Funding

The Data acquisition was supported by the ERA-Net Net‑Biome research framework, financed through the: Canary Islands Government ACIISI grants SE-12/02, SE-12/03, SE-12/04 co-financed by FEDER; Portuguese FCT-NETBIOME grant 0003/2011; French ANR-NETBIOME grant n°11-EBIM-001-01; Région Réunion council for research activities, Université de La Réunion contract DGADD/PE/20120585.

Darwin Core Database was implemented under the scope of the project Biodiversa+ project BioMonI – Biodiversity monitoring of island ecosystems and the Portuguese funding FCT – Fundação para a Ciência e a Tecnologia, BiodivMon/0003/2022, the French funding for the Agence Nationale de la Recherche, ANR-23-EBIP-0009-05 for the University of La Réunion, the Spanish funding for the University of La Laguna and Consejo Superior de Investigaciones Científicas, MCIN/AEI/10.13039/501100011033, project ID PCI2023-145966-2 and the Deutsche Forschungsgemeinschaft (DFG, German Research Foundation) — project ID 533271599 for University of Göttingen.

## Geographic coverage

### Description

This study was conducted in native, humid forest ecosystems with minimal anthropogenic influence across three oceanic islands of volcanic origin: Terceira, Tenerife and Réunion

### Coordinates

-21.341 and 39.266 Latitude; -27.773 and 55.802 Longitude.

## Taxonomic coverage

### Description

Kingdom: Plantae

Phylum: Magnoliophyta, Pinophyta, Pteridophyta.

Class: Magnoliopsida, Liliopsida, Pinopsida, Polypodiopsida.

Order: Apiales, Aquifoliales, Arecales, Asparagales, Asterales, Crossosomatales, Cyatheales, Dipsacales, Ericales, Fagales, Gentianales, Lamiales, Laurales, Magnoliales, Malpighiales, Malvales, Myrtales, Oxalidales, Pandanales, Pinales, Piperales, Rosales, Sapindales.

Family: Adoxaceae, Annonaceae, Apocynaceae, Aphloiaceae, Aquifoliaceae, Araliaceae, Arecaceae, Asparagaceae, Asteraceae, Calophyllaceae, Chrysobalanaceae, Connaraceae, Cunoniaceae, Cyatheaceae, Cupressaceae, Ericaceae, Euphorbiaceae, Lauraceae, Loganiaceae, Malvaceae, Melastomataceae, Meliaceae, Monimiaceae, Moraceae, Myricaceae, Myrsinaceae, Myrtaceae, Oleaceae, Pandanaceae, Pentaphylacaceae, Phyllanthaceae, Piperaceae, Primulaceae, Pittosporaceae, Rhamnaceae, Rosaceae, Rubiaceae, Rutaceae, Salicaceae, Sapindaceae, Sapotaceae, Stilbaceae.

## Temporal coverage

**Data range:** 2012-9-25 – 2013-7-24.

## Usage licence

### Usage licence

Creative Commons Public Domain Waiver (CC-Zero)

## Data resources

### Data package title

Plot Based biodiversity monitoring of trees on island ecosystems (Terceira, Tenerife and Réunion)

### Resource link

https://doi.org/10.15468/hwm3mt

### Alternative identifiers

http://ipt.gbif.pt/ipt/resource?r=biomoni_trees; https://www.gbif.org/dataset/eab982cf-81df-4d08-b9f4-c2f5c56b8ad3

### Number of data sets

2

### Data set 1.

#### Data set name

Event Table

#### Data format

Darwin Core Archive

#### Character set

UTF-8

#### Download URL

http://ipt.gbif.pt/ipt/resource?r=biomoni_trees

#### Data format version

1.6

#### Description

The dataset was published in the Global Biodiversity Information Facility platform, GBIF (Morgado et al. 2025). The following data table includes all the records for which a taxonomic identification of the species was possible. The dataset submitted to GBIF is structured as a sample event dataset that has been published as a Darwin Core Archive (DwCA), which is a standardised format for sharing biodiversity data as a set of one or more data tables. The core data file contains 30 records (eventID). This GBIF IPT (Integrated Publishing Toolkit, Version 2.5.6) archives the data and, thus, serves as the data repository. The data and resource metadata are available for download in the Portuguese GBIF Portal IPT (Morgado et al. 2025).

**Data set 1.** 

Column label	Column description
eventID	Identifier of the events, unique for the dataset.
locationID	An identifier specific to the dataset.
datasetName	The name identifying the dataset that in current projects is BIOMONI_ISLAND-BIODIV_AZO_Trees from Terceira Island, BIOMONI_ISLAND-BIODIV_CAN_Trees from Tenerife Island and BIOMONI_ISLAND-BIODIV_MAS_Trees from Réunion Island.
samplingProtocol	The sampling protocol used to survey woody species: Square plot of 50 m x 50 m.
eventDate	The date-time or interval during which an Event occurred.
day	The day of the month on which the Event occurred.
month	The month in which the Event occurred.
year	The year in which the Event occurred.
Habitat	Category or description of the habitat in which the Event occurred.
continent	The name of the continent in which the Location occurs (Europe and Africa).
islandGroup	The name of the island group in which the Location occurs (Azores, Canaries and Mascarene).
island	The name of the island on or near which the Location occurs (Terceira, Tenerife and Réunion).
country	The name of the country or major administrative unit in which the Location occurs (Portugal, Spain and France).
countryCode	The standard code for the country in which the Location occurs (PT, ES, FR).
municipality	The full, unabbreviated name of the next smaller administrative region than county (city, municipality etc.) in which the location occurs.
locality	The specific description of the place.
locationRemarks	Comments or notes on the Event location: priority areas.
minimumElevationInMetres	The original description of the elevation (altitude above sea level in metres) of the location.
verbatimCoordinates	Original coordinates recorded.
decimalLatitude	Approximate centre point decimal latitude of the field site in GPS coordinates.
decimalLongitude	Approximate centre point decimal longitude of the field site in GPS coordinates.
geodeticDatum	Standardised reference of the Global Positioning System coordinates of the plot location.
coordinateUncertaintyInMetres	Uncertain value of coordinate metrics.
coordinatePrecision	Value in decimal degrees to a precision of five decimal places.
georeferenceSources	Resources used to georeference the Event location.

### Data set 2.

#### Data set name

Occurrence Table

#### Data format

Darwin Core Archive

#### Character set

UTF-8

#### Download URL

http://ipt.gbif.pt/ipt/resource?r=biomoni_trees

#### Data format version

1.6

#### Description

The dataset was published in the Global Biodiversity Information Facility platform, GBIF (Morgado et al. 2025). The following data table includes all the records for which a taxonomic identification of the species was possible. The dataset submitted to GBIF is structured as an occurrence table that has been published as a Darwin Core Archive (DwCA), which is a standardised format for sharing biodiversity data as a set of one or more data tables. The core data file contains 402 records (occurrenceID). This GBIF IPT (Integrated Publishing Toolkit, Version 2.5.6) archives the data and, thus, serves as the data repository. The data and resource metadata are available for download in the Portuguese GBIF Portal IPT (Morgado et al. 2025).

**Data set 2.** 

Column label	Column description
eventID	Identifier of the events, unique for the dataset.
licence	Reference to the licence under which the record is published.
institutionID	The identity of the institution publishing the data.
institutionCode	The code of the institution publishing the data.
basisOfRecord	The specific nature of the data record that resulted from a process of human observation.
dynamicProperties	A list of additional measurements, facts, characteristics or assertions about the record, including IUCN categories (Endangered, Critically endangered, Least concern, Near threatened, Vulnerable) and colonisation status of taxa (Azorean endemic, Canary Endemic, Macaronesian endemic, Mascarene endemic, Non-endemic, Réunion endemic).
occurrenceID	Identifier of the record, coded as a global unique identifier.
recordedBy	A list of names of people, groups or organisations responsible for recording the original Occurrence.
identifiedBy	A list of names of people, who made the identification.
datasetName	Project reference: BIOMONI_ISLAND-BIODIV_AZO_Trees from Terceira Island, BIOMONI_ISLAND-BIODIV_CAN_Trees from Tenerife Island and BIOMONI_ISLAND-BIODIV_MAS_Trees from Réunion Island.
organismQuantityType	The type of counting system used for the quantity of organisms (individuals).
organismQuantity	A number or enumeration value for the quantity of organisms.
establishmentMeans	The process of establishment of the species in the location, using a controlled vocabulary: endemic, native, introduced.
dateIdentified	Date of species identification.
habitat	Category or description of the habitat in which the Event occurred.
kingdom	Kingdom name.
phylum	Phylum name.
class	Class name.
order	Order name.
family	Family name.
genus	Genus name.
specificEpithet	Specific epithet.
infraspecificEpithet	Infraspecific epithet at subspecies level.
scientificNameAuthorship	The authorship information for the scientificName formatted according to the conventions of the applicable nomenclaturalCode.
ScientificName	Complete scientific name including author.
taxonRank	Lowest taxonomic rank of the record.
cultivarEpithet	Part of the name of a cultivar, cultivar group or varietas that follows the scientificName.

## Additional information

A total of 4391 specimens were recorded in this study (2186 in Terceira; 714 in Tenerife and 1491 in Réunion), represented by 23 orders, 42 families, 69 genera and 93 taxa (species [n = 86], subspecies [n = 5], varietas [n = 1] and one taxon identified at the genus level) (Table 2). The phylum Magnoliophyta was the most represented (96.66%) of the total species and subspecies, followed by Pteridophyta (2.22%) and Pinophyta (1.11%). Réunion Island had the highest number of identified species (n = 66), compared to Tenerife (n = 16) and Terceira (n = 11). Only one species is shared between the studied islands, *Morellafaya*, that was found in Terceira and Tenerife. Notably, most of species were categorised (Colonisation Status) as endemic. Amongst these, 32 are Mascarene endemic, 22 are Réunion endemic, 11 are Macaronesian endemic, nine are Azorean endemic and four are Canary endemic. Additionally, eight endemic species are categorised (IUCN Status) as vulnerable and two are listed as endangered (Table 2). It is noteworthy that, within this study, of the total number of species on the three Islands, only one species (*Hederahelix* L.) is considered introduced (Tenerife Island), while the remaining species are classified as either endemic or native.

### Discussion and conclusions

The data presented in this Data Paper serve as a valuable proxy for assessing the overall habitat quality of native montane forests in the Azores, Canary and Mascarene archipelagos. By focusing on woody plants as primary ecological indicators, the dataset captures key structural and compositional attributes of these forest ecosystems, such as species richness and dominance expressed as basal area. Given that trees often form the foundational framework of forest habitats — shaping microclimates, influencing soil processes and providing essential resources for a wide array of flora and fauna — their characteristics offer meaningful insights into ecosystem integrity, successional stage and the degree of anthropogenic disturbance. As such, this information provides a critical baseline for long-term ecological monitoring, biodiversity assessments and conservation planning across these highly biodiverse and vulnerable island systems.

The establishment of monitoring plots enables the integration of multiple biodiversity components — plants, bryophytes, invertebrates and vertebrates — providing a holistic understanding of ecosystem health. For example, long-term plot-level data collected in the ten Terceira Island native montane Azorean forests, revealed the increase in diversity of exotic arthropods (Borges et al. 2020) and also a high temporal turnover of exotic arthropods (Matthews et al. 2019; Lhoumeau and Borges 2023). Similarly, a study using soil arthropod assemblages sampled in the 30 plots here described, showed that local-scale sampling reveals impacts of biological invasions by soil Collembola that might be missed with broader-scale surveys (Cicconardi et al. 2017). Moreover, surveys on those plots inspired the development of standardised protocols for molecular identification and monitoring of arthropods (Emerson et al. 2016, Emerson et al. 2022). Having plots for inventory and monitoring across archipelagos also allowed the inclusion of addtional plots in other Macaronesian islands and investigation of across-scale species turnover and rarity in island spider assemblages (Malumbres‐Olarte et al. 2021).

Moreover, an Index of Biotic Integrerity (IBI) was developed for Terceira Island plots using arthropod monitoring data (Tsafack et al. 2023), finding that the developed IBI values effectively differentiate between forest sites of varying ecological conditions. Higher IBI scores corresponded with areas exhibiting greater native biodiversity and lower levels of disturbance, while lower scores were associated with degraded habitats.

As proposed under Global Island Monitoring Scheme (Borges et al. 2018b), local monitoring plots can be standardised and scaled up into global networks to address broad questions about biodiversity loss, climate change impacts and conservation outcomes across islands worldwide.

In parallel, the establishment of BioMonI-Plot, a standardised network of vegetation plots, facilitates the in-depth study of biodiversity and ecosystem change by providing spatially and temporally explicit data on species composition and ecological processes. Finally, within the scope of EU BIODIVERSA + project BioMonI, we aim at scaling up biodiversity monitoring to regional and global levels requiring integrating remote sensing technologies, macroecological modelling and scenario-based forecasting. These tools, combined with a future BioMonI E-intrastructure (BioMonI Portal) will allow a broad set of stakeholders to assess ecosystem structure and function at broad spatial scales and to predict future changes under different environmental and land-use scenarios. Together, these approaches form a comprehensive framework for understanding and managing biodiversity in a rapidly changing world.

## Supplementary Material

D6FB058D-B4E8-55BA-842E-79A5223F5837 10.3897/BDJ.13.e158423.suppl1 Supplementary material 1 Mean DBH (for shoots > 10 cm) for each plant species in the ten plots of Terceira Island (Azores) Data type Mean values of DBH Brief description For each plant species with shoots > 10 cm, the mean value of DBH is listed for each of the 10 plots. File: oo_1325162.csv https://binary.pensoft.net/file/1325162 Rui B. Elias

F702776D-97F1-59B7-A35A-ABBA9CEFC402 10.3897/BDJ.13.e158423.suppl2 Supplementary material 2 Mean DBH (for shoots > 10 cm) for each plant species in the ten plots of Tenerife Island (Canary Islands) Data type Mean values of DBH Brief description For each plant species with shoots > 10 cm, the mean value of DBH is listed for each of the 10 plots. File: oo_1325163.csv https://binary.pensoft.net/file/1325163 José María Fernandez-Palácios

D69D7D96-E89A-5CEB-9AA0-57260F3728AD 10.3897/BDJ.13.e158423.suppl3 Supplementary material 3 Mean DBH (for shoots > 10 cm) for each plant species in the ten plots of Réunion Island (Mascarenes) Data type Mean values of DBH Brief description For each plant species with shoots > 10 cm, the mean value of DBH is listed for each of the 10 plots. File: oo_1325164.csv https://binary.pensoft.net/file/1325164 Dominique Strasberg

## Figures and Tables

**Table 1.** Data on the locations of woody plant samples from the Islands of Terceira, Tenerife and Réunion, including plot codes, locationID, locality, elevation (m a.s.l.) and coordinates (decimal degrees).

Island	Plot Code	locationID	Locality	Elevation (m a.s.l.)	decimalLatitude	decimalLongitude
Terceira	AZO_TER_PLOT_01	TER_NFBF_T01	Labaçal - Morro Assombrado	694	38.7618	-27.2193
	AZO_TER_PLOT_02	TER_NFBF_T02	Chambre A	575	38.7521	-27.2331
	AZO_TER_PLOT_03	TER_NFBF_TP41	Pico Alto Nascente	686	38.7502	-27.2072
	AZO_TER_PLOT_04	TER_NFPG_T33	Pico X B	651	38.7334	-27.2271
	AZO_TER_PLOT_05	TER_NFSB_T07	Lomba	693	38.7372	-27.2899
	AZO_TER_PLOT_06	TER_NFSB_T164	Caldeira - Silvia	890	38.7355	-27.3074
	AZO_TER_PLOT_07	TER_NFSB_TE48	Lagoinha B	748	38.7521	-27.3313
	AZO_TER_PLOT_08	TER_NFSB_TE49	Lagoa Pinheiro B	930	38.7471	-27.3196
	AZO_TER_PLOT_09	TER_NFTB_T15	Terra Brava - A	639	38.7364	-27.2006
	AZO_TER_PLOT_10	TER_NFTB_T18	Terra Brava - B	668	38.7323	-27.1980
Tenerife	CAN_TEN_PLOT_11	TEN_AGU	Monte Aguirre	861	28.5329	-16.2696
	CAN_TEN_PLOT_12	TEN_ANE	Aguas Negras	866	28.5398	-16.2247
	CAN_TEN_PLOT_13	TEN_CHI	Chinobre	870	28.5588	-16.1737
	CAN_TEN_PLOT_14	TEN_CTE	Cabezo del Tejo	851	28.5621	-16.1722
	CAN_TEN_PLOT_15	TEN_IJU	Hoya de Ijuana	772	28.5606	-16.1697
	CAN_TEN_PLOT_16	TEN_MOQ	El Moquinal	772	28.5366	-16.3088
	CAN_TEN_PLOT_17	TEN_NIE	Bco. de Nieto	771	28.5315	-16.3185
	CAN_TEN_PLOT_18	TEN_PIJ	Pijaral	792	28.5524	-16.1892
	CAN_TEN_PLOT_19	TEN_TAG	Vueltas de Taganana	840	28.5439	-16.2261
	CAN_TEN_PLOT_20	TEN_ZAP	Zapata	890	28.5358	-16.2962
Réunion	MAS_REU_PLOT_21	REU_TB01	Mare Longue	580	-21.3412	55.7398
	MAS_REU_PLOT_22	REU_TB02	Basse Vallee	769	-21.3386	55.7093
	MAS_REU_PLOT_23	REU_TB03	Piton Nelson	679	-21.2026	55.7882
	MAS_REU_PLOT_24	REU_TB04	Piton la Glace	487	-21.1733	55.8022
	MAS_REU_PLOT_25	REU_TB05	Riviere de l'Est	651	-21.1446	55.7379
	MAS_REU_PLOT_26	REU_TB06	Intermediaire Basse Vallée	692	-21.3382	55.7175
	MAS_REU_PLOT_27	REU_TB07	Sainte Marguerite	676	-21.1141	55.6796
	MAS_REU_PLOT_28	REU_TB08	Grand Etang	575	-21.0941	55.6478
	MAS_REU_PLOT_29	REU_TB09	Cascade du Chien	791	-21.0358	55.6056
	MAS_REU_PLOT_30	REU_TB10	Bras Laurent	795	-20.9814	55.5641

**Table 2.** List of species, subspecies and varietals sampled on the three Islands (Terceira (TER), Tenerife (TEN) and Réunion (REU), with their respective colonisation status categories and IUCN Status.

Scientific Name	Biogeographical status	IUCN Status	Island
*Acalyphaintegrifolia* Willd.	Mascarene endemic	Least concern	REU
*Acanthophoenixrubra* (Bory) H.Wendl.	Mascarene endemic	Endangered	REU
*Agaristasalicifolia* (Lam.) G.Don	Non-endemic	Least Concern	REU
*Allophylusborbonicus* (J.F.Gmel.) F.Friedmann	Mascarene endemic	Least concern	REU
*Alsophilaborbonica* (Desv.) R.M.Tryon	Mascarene endemic	Least concern	REU
*Alsophilacelsa* R.M.Tryon	Mascarene endemic	Least concern	REU
*Antidesmamadagascariense* Lam.	Non-endemic	Least Concern	REU
*Antirheaborbonica* J.F.Gmel.	Non-endemic	Least Concern	REU
*Aphloiatheiformis* (Vahl) Benn.	Non-endemic	Least Concern	REU
*Badulabarthesia* (Lam.) A.DC.	Réunion endemic	Least concern	REU
*Badulaborbonica* A.DC.	Réunion endemic	Least concern	REU
*Badulagrammisticta* (Cordem.) Coode	Réunion endemic	Least concern	REU
*Badulanitida* (Coode) Coode	Réunion endemic	Vulnerable	REU
*Bremerialandia* (Poir.) Razafim. & Alejandro	Mascarene endemic	Least concern	REU
*Callunavulgaris* (L.) Hull	Non-endemic	Least Concern	TER
*Calophyllumtacamahaca* Willd.	Mascarene endemic	Near threatened	REU
*Caseariacoriacea* Vent.	Mascarene endemic	Least concern	REU
*Chassaliacorallioides* (Cordem.) Verdc.	Reunion endemic	Least concern	REU
*Chassaliagaertneroides* (Cordem.) Verdc.	Réunion endemic	Least concern	REU
*Cnestisglabra* Lam.	Non-endemic	Least Concern	REU
*Coffeamauritiana* Lam.	Mascarene endemic	Vulnerable	REU
*Cordylinemauritiana* (Lam.) J.F.Macbr.	Mascarene endemic	Least concern	REU
*Danaisfragrans* (Lam.) Pers.	Non-endemic	Least Concern	REU
*Dombeyaciliata* Cordem.	Réunion endemic	Least concern	REU
*Dombeyaelegan*s Cordem.	Non-endemic	Least Concern	REU
*Dombeyaficulne*a Baill.	Réunion endemic	Least concern	REU
*Doratoxylonapetalum* (Poir.) Radlk.	Non-endemic	Least Concern	REU
*Embeliaangustifolia* (A.DC.) A.DC.	Mascarene endemic	Least concern	REU
*Ericaazorica* Hochst. ex Seub.	Azorean endemic		TER
*Ericacanariensis* Rivas-Mart., M. Osorio & Wildpret	Macaronesian Endemic		TEN
Ericaplatycodon(Webb & Berthel.)Rivas-Mart. & al.subsp.platycodon	Canary Endemic		TEN
*Ficuslateriflora* Vahl	Mascarene endemic	Critically endangered	REU
*Ficusmauritiana* Lam.	Mascarene endemic	Least concern	REU
*Frangulaazorica* Grubov	Azorean endemic	Least concern	TER
*Gaertneravaginata* Poir.	Réunion endemic	Least concern	REU
*Geniostomaborbonicum* Spreng.	Mascarene endemic	Least concern	REU
*Grangeriaborbonica* Lam.	Mascarene endemic	Least concern	REU
*Gymnanthemumfimbrilliferum* Cass.	Réunion endemic	Least concern	REU
*Hanceaintegrifolia* (Willd.) S.E.C.Sierra, Kulju & Welzen	Mascarene endemic	Least concern	REU
*Heberdeniaexcelsa* (Aiton) Banks ex DC.	Macaronesian endemic	Vulnerable	TEN
*Hederahelix* L.	Non-Endemic		TEN
*Hibiscusboryanus* DC.	Mascarene endemic	Least concern	REU
*Homaliumpaniculatum* (Lam.) Benth.	Mascarene endemic	Least concern	REU
*Hubertiaambavilla* Bory	Mascarene endemic	Least concern	REU
*Ilexazorica* Gand.	Azorean endemic	Least concern	TER
*Ilexcanariensis* Poir.	Macaronesian endemic	Least concern	TEN
*Ilexperado* Aiton subsp. platyphylla	Canary endemic	Least Concern	TEN
Juniperusbrevifolia(Hochst. ex Seub.)Antoinesubsp.brevifolia	Azorean endemic	Vulnerable	TER
*Labourdonnaisiacalophylloide*s Bojer	Mascarene endemic	Least concern	REU
*Laurusazorica* (Seub.) Franco	Azorean endemic	Least concern	TER
*Laurusnovocanariensis* Rivas-Mart., Lousa, Fern. Prieto, E. Días, J.C. Costa & C. Aguiar	Macaronesian endemic	Least concern	TEN
*Maillardiaborbonica* Duch.	Réunion endemic	Least concern	REU
Melicopeborbonicavar.acuminata (Coode) T.G.Hartley	Réunion endemic		REU
*Melicopeobscura* (Cordem.) T.G.Hartley	Réunion endemic	Least concern	REU
*Memecylonconfusum* Blume	Réunion endemic	Least concern	REU
*Memecyloncordatu*m Lam.	Mascarene endemic	Endangered	REU
*Mimusopsbalata* (Aubl.) C.F.Gaertn.	Mascarene endemic	Least concern	REU
*Molinaeaalternifolia* Willd.	Mascarene endemic	Least concern	REU
*Monimiaovalifoli*a Thouars	Mascarene endemic	Least concern	REU
*Morellafaya* (Aiton) Wilbur	Macaronesian endemic	Least concern	TER | TEN
*Myrsineretusa* Aiton	Azorean endemic		TER
*Noronhiabroomeana* Horne ex Oliv.	Mascarene endemic	Least concern	REU
*Nuxiaverticillata* Lam.	Mascarene endemic	Least concern	REU
*Ocoteafoetens* (Aiton) Baill.	Macaronesian endemic	Least Concern	TEN
*Ocoteaobtusata* (Nees) Kosterm.	Mascarene endemic	Least concern	REU
*Pandanusmontanus* Bory	Réunion endemic	Least concern	REU
*Pandanuspurpurascens* Thouars	Réunion endemic	Least concern	REU
*Perseabarbujana* (Cav.) Mabb. & Nieto Fel.	Macaronesian endemic	Least concern	TEN
*Perseaindica* (L.) Spreng.	Macaronesian endemic	Least concern	TEN
*Phyllanthusphillyreifolius* Poir.	Réunion endemic		REU
*Picconiaazorica* (Tutin) Knobl.	Azorean endemic	Least concern	TER
*Picconiaexcelsa* (Aiton) DC.	Macaronesian endemic	Least Concern	TEN
*Piperborbonens*e (Miq.) C.DC.	Non-endemic	Least Concern	REU
*Pittosporumsenacia* Putt.	Non-endemic	Least Concern	REU
*Polysciasrepanda* (DC.) Baker	Réunion endemic	Least concern	REU
*Prunuslusitanica* L. subsp. hixa	Macaronesian endemic	Least Concern	TEN
*Psiloxylonmauritianum* (Bouton ex Hook.fil.) Baill.	Mascarene endemic	Least concern	REU
*Rubusbollei* Focke	Canary Endemic	Least concern	TEN
*Syzygiumborbonicum* J.Guého & A.J.Scott	Réunion endemic	Vulnerable	REU
*Syzygiumcordemoyi* Bosser & Cadet	Réunion endemic	Least concern	REU
*Syzygiumcymosum* (Lam.) DC.	Mascarene endemic	Least concern	REU
*Tabernaemontanamauritiana* Poir.	Mascarene endemic	Near threatened	REU
Tambourissaellipticasubsp.micrantha Lorence	Réunion endemic		REU
*Turraeacadetii* A.J.Scott	Réunion endemic	Vulnerable	REU
*Turraeaovata* (Cav.) Harms	Mascarene endemic	Vulnerable	REU
*Vacciniumcylindraceum* Sm.	Azorean endemic	Least concern	TER
*Viburnumrugosum* Pers.	Canary Endemic	Least concern	TEN
*Viburnumtreleasei* Gand.	Azorean endemic	Least concern	TER
*Visneamocanera* L. f.	Macaronesian endemic	Least concern	TEN
*Weinmanniatinctoria* Sm.	Non-Endemic	Critically endangered	REU
*Xylopiarichardii* Boivin ex Baill.	Mascarene endemic	Vulnerable	REU
*Zanthoxylumasiaticum* (L.) Appelhans, Groppo & J.Wen	Non-endemic	Least Concern	REU
